# Centennial-scale gaps in a 5500-year acroporid growth trajectory from a Caribbean coral reef

**DOI:** 10.1098/rsos.250363

**Published:** 2025-07-30

**Authors:** Alexis Medina-Valmaseda, Paul Blanchon, Juan Pablo Bernal, Edlin Guerra-Castro, Liliana Corona-Martinez, Alexander Correa-Metrio

**Affiliations:** ^1^ Instituto de Geociencias, Universidad Nacional Autónoma de México, Querétaro, Mexico; ^2^ Instituto de Ciencias del Mar y Limnología, Universidad Nacional Autónoma de México, Puerto Morelos, Quintana Roo, Mexico; ^3^ Escuela Nacional de Estudios Superiores Unidad Merida, Universidad Nacional Autónoma de México, Merida, Yucatan, Mexico

**Keywords:** coral reefs, palaeoecology, Caribbean, Holocene, growth trajectory, hurricanes, extirpation, recolonization, acroporids, global warming

## Abstract

Persistence of acroporid-dominated assemblages on Caribbean reefs throughout the Holocene and late Pleistocene implies that their rapid regional demise over the last 50 years is unprecedented. However, the palaeoecological trajectory of acroporid growth is largely unknown. Here, we reconstruct a 5500-year acroporid trajectory from a hurricane-prone fringing reef off the northeast Yucatan coast and find that growth is not constant but punctuated by centennial-scale gaps. Local coastal archives show these gaps coincide with hurricane-frequency anomalies, which is consistent with local extirpation of acroporids following intense hurricane strikes. On each devastated reef, acroporids took hundreds of years to recolonize their former habitat, probably owing to naturally impaired sexual recruitment combined with substrate deterioration. By comparing trajectories across the Caribbean, we show that extirpation-recolonization events occur at different times between reefs, so gaps do not coincide. The resulting regional constancy of this palaeoecological baseline affirms that the historical demise of acroporids is unprecedented over the last 14 000 years and portends their absence on degraded reefs for hundreds of years into the future unless mitigated by restoration.

## 1. Introduction

Caribbean reefs were once covered by dense and diverse communities of corals [1–4]. The first underwater surveys reported lush coral cover with particularly dense thickets of robust-branching *Acropora palmata* over the shallow crest and reef-front zones [5,6]. The dominance of *A. palmata* in these wave-swept habitats stemmed from their large size and close colony spacing, which produced interlocking surf-resistant thickets capable of absorbing wave energy and modulating the surrounding environment [6,7]. Their competitive success is attributed to a 5−10 cm yr^−1^ growth rate, which is an order of magnitude higher than other reef-building species, and efficient reproduction via asexual fragmentation and re-sheeting of dead colonies [8–10]. These traits allow a quick recovery from storm damage, potentially creating a continuous cycle of disturbance and recovery over ecological timescales [10–12]. Such a cycle would also be consistent with the geological structure of both Holocene and Pleistocene reef-crest units, many of which are composed predominantly of hurricane-generated acroporid clasts [13–15].

Today, these reef-crest habitats are devoid of large corals owing to the widespread mortality of acroporids and steep decline in coral cover over the last 50 years [16–19]. None of the degraded reefs have subsequently shown significant evidence of recovery, apparently owing to the absence of recruitment [12,20,21]. Given their ecological and geological importance, this lack of recovery has not only transformed shallow reefs into less complex habitats dominated by non-reef-building species [16,17] but has also led to widespread changes in reef structure and function, which threaten to impair their future development [22,23].

The direct cause of the regional decline in Caribbean acroporids is widely attributed to the combination of increased disease outbreaks and hurricane intensity [3,24–26]. However, the proximate cause is contested, involving either local chronic anthropogenic disturbance preventing recovery, or a regional increase in sea-surface temperature rendering coral populations susceptible to disease and bleaching [27–29]. Regardless of the cause, the historical demise of acroporids over the last 50 years is considered unprecedented on millennial timescales [1–4,30]. This claim is based on the compositional consistency between modern and fossil reef communities, which implies that acroporid dominance has been the regional baseline since the beginning of the Pleistocene [1,30]. Despite this apparent community consistency, few studies have sought to establish the palaeoecological growth trajectory of acroporid assemblages in fossil reefs to determine if there have been comparable widespread die-offs in the past. Yet the degree of constancy in past acroporid growth is key to understanding the cause of its historical demise, especially given that both the late Pleistocene and Holocene have encompassed significant intervals of natural climatic variation [31–33]. Although the impact of local anthropogenic activity on reef decline is undeniable [34], the widespread demise of reefs in sparsely populated areas [35] necessitates differentiating the proximate cause of regional decline from natural variation.

To determine the constancy of acroporid growth on millennial timescales, we reconstruct its trajectory on a fringing reef at Punta Maroma, along the northeast Yucatan Peninsula, Mexico. Given its tectonic stability [36,37] and riverless karstic terrain, the Yucatan is optimal for long-term reef development. Furthermore, the geological structure of the reef at Punta Maroma has been reconstructed from a U-series-dated core transect, showing it consists of a deposit of hurricane-generated acroporid clasts that retrograded shoreward over the last 5500 years, driven by hurricanes and sea-level rise [15]. Lastly, the historical growth of shallow acroporids has been monitored since the late 1970s, showing a similar decline to other reefs in the region [9]. From these data, we assume that acroporid growth and post-mortem clast transport have been retained within the crest and reef-front zones, given that previous surveys report a 5 m depth limit for the *A. palmata* zone [9]. Although the geomorphic boundary of the reef-front zone extends to a maximum depth of 8 m, this larger depth interval is still consistent with the acroporid growth habitat because sea level in the Caribbean has risen approximately 3 m over the last 5500 years [15]. Consequently, sampling these zones should provide a representative picture of the acroporid growth trajectory over this time interval.

## 2. Methods

### 2.1. Near-surface sampling

**Figure 1.**
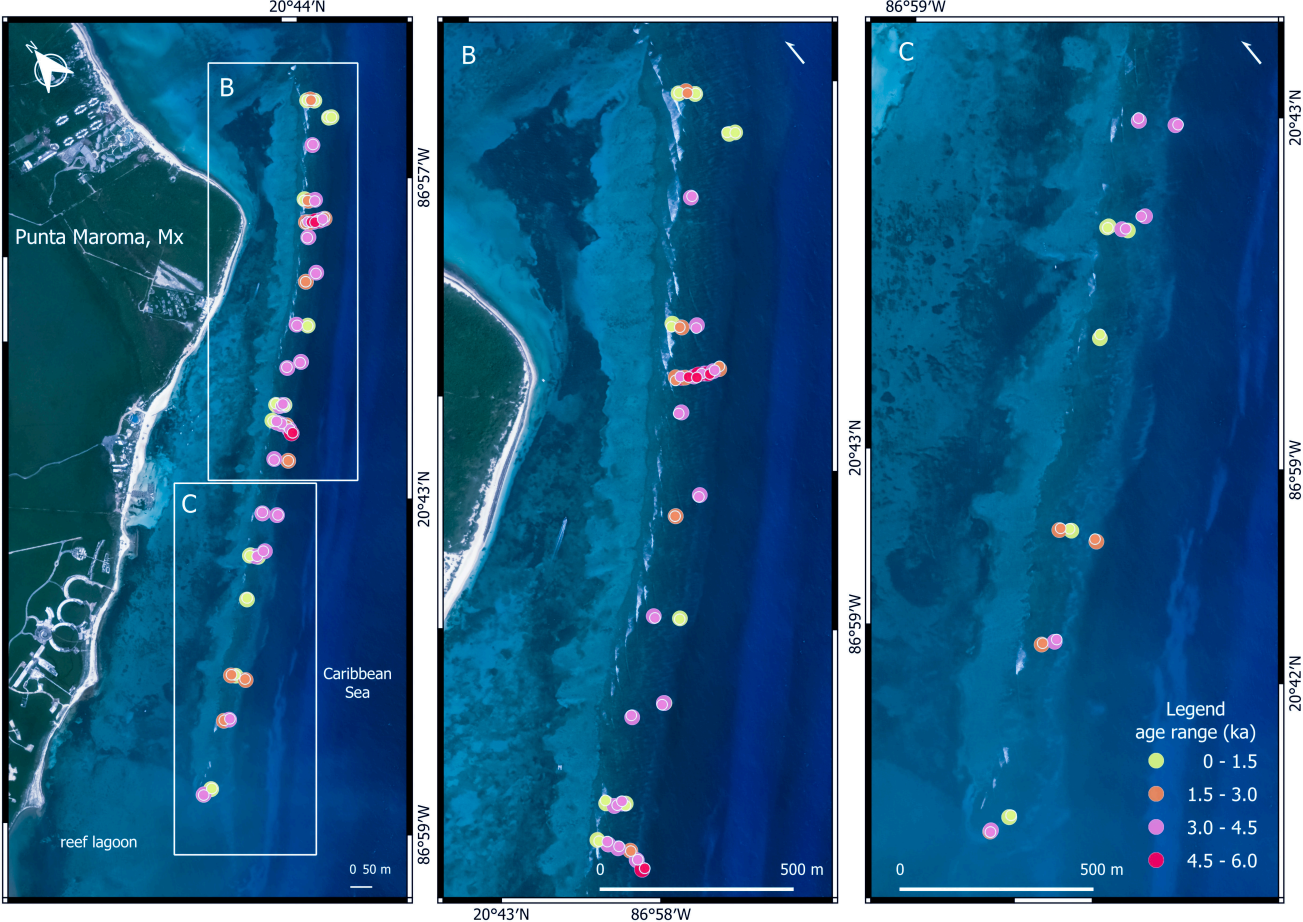
Spatial distribution of near-surface ages at Punta Maroma. Circle colour represents the age range (ka) for each site: yellow <1.5 ka; orange, 1.5−3.0 ka; pink, 3.0−4.5 ka and red, 4.5−6.0 ka. Esri Imagery 2D (Maxar, Earthstar Geographics and the GIS user company) was accessed through Qgis plugin HCMGIS (GitHub - thangqd/HCMGIS: HCMGIS Plugin for QGIS), freely distributed under GNU General Public License v3.0. Geographical coordinates are represented in UTM 16N (Mexico).

To determine the chronological trajectory of acroporid growth at Punta Maroma, we sampled the near-surface substrate of the crest and reef-front zone, where ecological surveys show past acroporid dominance [9] (electronic supplementary material, S1). In the summers of 2019 and 2021, we collected coral clasts from shallow sub-meter-sized pits excavated into the stabilized, but largely uncemented substrate of the crest and reef-front (figure 1; electronic supplementary material, figure S5). The selection of sample sites was based on the need to cover both lateral and downslope extents of these zones across the entire 4 km long reef structure. For this reason, we collected samples along several transect-like traverses perpendicular to the crest and more isolated single sites distributed haphazardly along the reef-front. In total, 52 pits were excavated (electronic supplementary material, S2). In each, we prioritized the collection of large acroporid species in order to reconstruct their trajectory. However, we also collected a lesser number of non-acroporid species to determine the degree of community consistency (samples were identified to species level where possible). In addition to pit samples, we include 13 near-surface acroporid samples from 10 cores of the 12-core drill transect, with nine samples taken from the top 1 m of each core, and four samples between 1 and 2 m of the surface [15]. Including near-surface samples from both pits and cores gives a total of 62 sampling sites distributed non-uniformly over a reef area of approximately 0.65 km^2^ (figure 1).

### 2.2. Geochronology

Coral samples from the pits and cores were screened for dating both visually, based on the skeletal preservation state, and isotopically based on the ^238^U concentration of modern coral species (2.0−3.5 p.p.m.), and the ^232^Th concentration of 2 p.p.b. Screened coral samples were dated using ^234^U/^230^Th radioisotope series at the Laboratorio de Estudios Isotópicos, Centro de Geociencias, UNAM, using a multi-collector ICPMS Thermo Neptune Plus mass spectrometer. All sample preparations were carried out in Class 100 Clean Laboratory, with typical blanks of less than 3 pg of ^238^U and 1 pg of ^232^Th. The process involved using high-purity ^233^U and ^229^Th spikes, calibrated against solutions of uraninite in secular equilibrium (Harwell Uraninite and Schwartzwalder Mine Uranium Mineral). The dating methodology corresponds to U and Th separation and purification reported by [15,38]. The activity ratios were calculated considering the decay constants for ^230^Th and ^234^U reported by [39], for ^238^U reported by [40] and for ^232^Th reported by [41]. The raw ages were corrected for detrital Th contribution using a two-point isochron with the atomic ratio of ^232^Th/^238^U of the detrital fraction assumed to be similar to that of the Earth’s crust = 3.8 ± 1.2 [42], and with [^230^Th/^238^U] and [^234^U/^238^U] equal to 1.0 ± 0.1 [43]. The same constants were used for age calculation using Isoplot-R [44]. Repeat analyses of a mid-Holocene coral sample yield an average U-Th age of 4.59 ± 0.06 (2 s.e., *n* = 10, mean square weighted deviation 5.5) within different analytical sessions. Neither the analytical error of measured age nor the true-age variation between duplicate ages exceeded 1%. All ages and their corresponding uncertainties are represented in absolute calendar years from the moment of U-Th separation (2021 and 2023) following [45]. The U/Th activity ratios and 230Th-ages for coral samples from Punta Maroma generated in this study are provided in the electronic supplementary material, table S1. All acroporid ages returned delta ^234^U initial ratios and ^238^U concentrations consistent with modern seawater and coral values, respectively.

### 2.3. Statistical analysis

Given that the production of coral clasts at Punta Maroma is related to multiple cycles of disturbance and recovery over the last 5500 years [15], it is reasonable to assume that their age distribution will exhibit more stochastic patterns, such as multiple modes or discontinuities. Consequently, to assess the integrity of our age distribution, we perform three analyses (electronic supplementary material, S3): we first construct a frequency distribution of ages in 100-year intervals and use a kernel density estimate (KDE) to show distribution patterns. Then we build a null model based on Monte Carlo simulations to determine the probability of observing gaps in a theoretical distribution parameterized with the acroporid data [46]. Finally, we assess data sufficiency with a resampling approach to determine if the sample size affects the age distribution [47].

**Figure 2.**
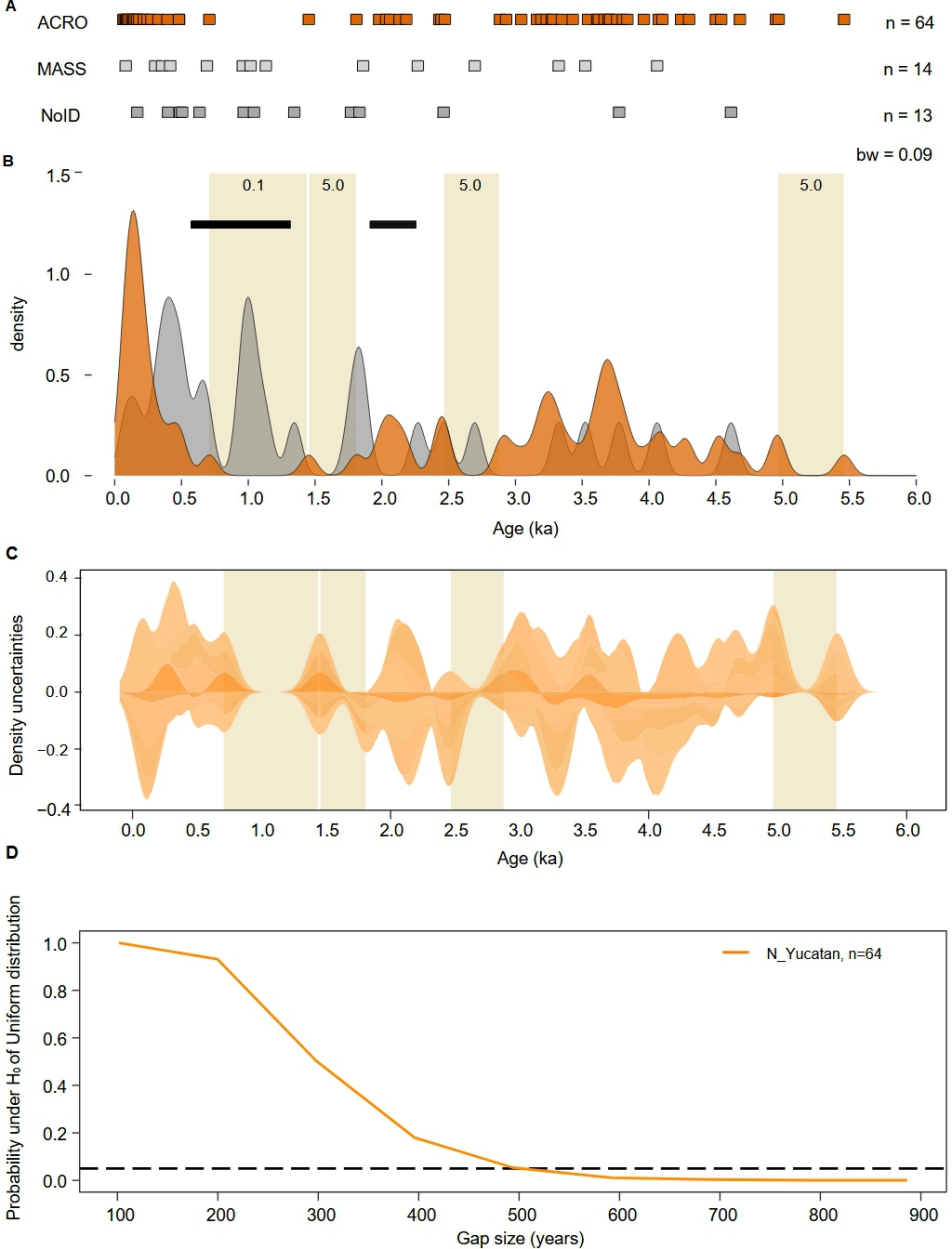
Gaps in acroporid growth trajectory from Punta Maroma. (A) Near-surface U-series ages in calendar years arranged into three groups: ACRO (orange) branching *A. palmata* (*n* = 61) and *Acropora cervicornis* (*n* = 3), MASS (light grey) massive and submassive species (*n* = 14) and NoID (dark grey) non-acroporid samples without reliable identification (*n* = 13). Age uncertainties are smaller than the size of the box symbol used (electronic supplementary material, table S1). (B) Density plot (kernel density estimates) showing periods of acroporid growth in orange separated by four centennial-scale gaps (0.71−1.45, 1.45−1.81, 2.47−2.88, 4.97−5.46 ka) highlighted with vertical bands. Each band shows the probability of occurrence (%) estimated from random sampling of a uniform null-model distribution. Non-acroporid growth in grey (combining MASS and NoID) fills acroporid gaps. The black bars represent intervals of anomalously high hurricane frequency recorded from Laguna Muyil, the closest coastal-sediment record to Punta Maroma. (C) Uncertainties associated with gaps in acroporid age data (vertical bands) from Punta Maroma. Uncertainties (95% confidence interval) cover three of the four gaps, indicating only the youngest gap is statistically robust. Calculated bandwidth is similar in extension and close to a centennial value (0.088 and 0.096). (D) Monte Carlo simulation of the probability of centennial-scale gaps under a uniform null-model distribution parameterized with the 64 acroporid samples. The dashed horizontal line shows the statistical threshold of *p* = 0.05 (5%). The results indicate that gaps greater than approximately 500 years are statistically improbable.

To find an appropriate theoretical distribution for the Monte Carlo simulation, we first fit all age data (both acroporids and non-acroporids) to several continuous distributions (i.e. uniform, lognormal, exponential, gamma; electronic supplementary material, figure S2 and table S4). The fit considers two situations: (i) the entire range of ages, and (ii) a forced exclusion of the most recent 500 years. In both situations, the fitting-model analysis is based on two common quality evaluation criteria: the Akaike information criterion and the Bayesian information criterion. Then, we employ the Monte Carlo simulation to estimate the likelihood of observing gaps of different sizes in a uniform distribution parameterized with the acroporid data (i.e. sample size, minimum and maximum values), sampling random values in a procedure that is repeated 10 000 times. For each simulation, we use sample size, the number of gaps of specific size and the frequency of occurrence to estimate the probability of finding a specific gap size by chance. We then test the null hypothesis of finding gap sizes of 50, 100, 150 years, until 950 years by chance, by comparing it with the simulated data using a threshold value of 0.05 or 5% (figure 2D; electronic supplementary material, figure S3).

Finally, to assess data sufficiency, we employ a resampling approach to determine if the sample size affects the age distribution [47]. It calculates uncertainties associated with data distribution for various sample sizes and plots absolute differences between these and the age data (electronic supplementary material, figure S4). Resampling is also used to assess the reliability of discontinuities in the data distribution by constructing a 95% confidence interval and corresponding inter-quantile ranges, producing a computed KDE age distribution of means using the unbiased cross validation function to calculate KDE bandwidth (figure 2C). This plot encompasses 1000 resampling of 40 randomly selected data points from the age data. All analyses and visualizations are performed with the statistical software R [48]. All data and codes are available at: https://github.com/AlexisMedina2019/acroporid-centennial-gap-analysis.

## 3. Results

### 3.1. Chronology of acroporid growth

The near-surface U-series age (in calendar years) of the reef is based on 101 skeletal clasts collected from 62 sites across the crest and reef-front zones (electronic supplementary material, table S1). Ten samples returned ages between 30 000 and 109 000 years, representing polycyclic clasts either eroded from the underlying substrate or reworked from relict coastal deposits during the Holocene transgression. As such, these samples are excluded from subsequent growth-trajectory analysis. The remaining 91 clasts returned Holocene ages ranging from 5460 ± 60 to 60 ± 0.1 years, affirming the age of reef development at Punta Maroma [15] and yielding a palaeoecological resolution of approximately 60 years. Among this total, 64 clasts correspond to the genus *Acropora* (61 identified as *A. palmata* and three as *Acropora cervicornis*), while 27 belong to non-Acroporid species. Of these, only 14 could be clearly identified: *Siderastrea siderea* (5), *Orbicella faveolata* (3), *Porites astreoides* (2)*, Pseudodiploria strigosa* (2)*, Dendrogyra cylindrus* (1) and *Colpophyllia natans* (1). The remaining 13 clasts were identified as non-acroporids but could not be further differentiated to species level.

The spatial distribution of clast ages across the reef and within individual sites is heterogeneous, consistent with mixing of different generations of acroporids through time [15], but inconsistent with an *in-situ* framework of progressively younger corals (figure 1). Three main age groups are shown by the KDE of acroporid growth trajectory over the last 5.5 ka (figure 2): in the first group, 36% of growth occurs in the last 700 years, in the second, 14% of growth occurs between 1800 and 2400 cal. years, and in the third, 48% of growth occurs between 2800 and 5000 cal. years. The percentage of growth in these age groups is unlikely to be representative given the unequal surface-residence times and thus degree of mixing.

Separating these growth groups are four centennial-scale gaps spanning 360−740 ± 30 cal. years (figure 2). The first two gaps (740 and 360 years) occur between groups 1 and 2, from 700 to 1800 cal. years but are separated by only a single age. The third gap of 400 years occurs between groups 2 and 3, from 2500 to 2900 cal. years, and the last gap of approximately 500 years separates group 3 from the oldest acroporid age (figure 2).

In contrast to the heterogeneous acroporid trajectory, non-acroporid ages are more uniformly spread over the 5.5 ka interval of reef development. Although there are insufficient samples to determine a representative growth trajectory, it is apparent that non-acroporid growth fills gaps in the acroporid record, implying that non-acroporid corals grew during times when acroporids were apparently absent.

### 3.2. Significance of gaps

The probability of encountering centennial-scale gaps in the acroporid trajectory is simulated by a null-model Monte Carlo analysis parameterized with the 64 acroporid samples (figure 2D). This simulation shows that gaps of 500 years can occur with a 5% probability, but larger gaps are improbable (see also the electronic supplementary material, figure S3). As a result, only the largest 740-year gap in the acroporid growth trajectory, with an anomalously low chance of 0.13%, is consistent with acroporid absence (figure 2). The uncertainty of these probability estimates is assessed by comparing a KDE of the real-age distribution to resampled data (figure 2C). Near equivalency is achieved with 35 samples, confirming that 64 samples are sufficient to accurately characterize the trajectory of acroporids at Punta Maroma (electronic supplementary material, figure S4A). Further resampling also confirms that the 740-year gap is the only one uncovered by the 95% confidence interval in KDE density uncertainty (figure 2C).

### 3.3. Role of hurricanes

The presence of statistically anomalous centennial-scale gaps in the trajectory of acroporid growth at Punta Maroma helps eliminate short-term ecological disturbance as a cause. For example, acute disturbances like disease outbreaks or bleaching, typically see recovery on a sub-decadal scale. This rapid recovery is possible because some corals survive and standing-dead skeletons facilitate re-sheeting and recruitment [49]. Hurricanes are also acute disturbances, and less intense strikes can again leave survivors and varying degrees of skeletal damage leading to decadal recovery intervals [2]. None of these types of acute disturbance can account for the absence of coral growth over seven centuries: this requires an exceptional event capable of exterminating live acroporids, destroying their skeletons and circumventing recovery from local sources. Such events are rare on a stable and riverless Peninsula, with the exception of strikes from particularly intense hurricanes or multiple successive strikes from less intense hurricanes. Indeed, anomalies in hurricane frequency arise stochastically over time [50], which could account for such long gaps in acroporid trajectories.

To test the hypothesis that long gaps result from intense or frequent hurricanes, we compare the growth trajectory of acroporids at Punta Maroma with an independent reconstruction of hurricane frequency from a nearby coastal sediment archive. This archive is from Laguna Muyil, approximately 80 km south of Punta Maroma [51] and spans the last 2 ka. It shows two intervals with hurricane-frequency anomalies: persistent below-average frequency between 300 and 550 cal. years ago and persistent above-average frequency between 550 and 1300 cal. years ago (figure 2B). These frequency anomalies coincide with acroporid growth at Punta Maroma, with growth-group 1 corresponding to reduced hurricane frequency between 300 and 550 cal. years ago, and the gaps between 700 and 1800 cal. years coinciding with increased hurricane frequency between 550 and 1300 cal. years (figure 2B). However, a brief interval of frequent hurricanes from 2.25 to 1.90 ka aligns with acroporid growth, showing that, without an intensity metric, it is difficult to fully assess hurricane impact on acroporid growth.

## 4. Discussion

Despite the uncertainty of matching hurricanes with acroporid trajectories, the presence of centennial-scale gaps at Punta Maroma implies that acroporid growth was suppressed for hundreds of years. This is a significant departure from the paradigm of ecological constancy and a continuous cycle of disturbance and recovery [2], which is assumed to have been the baseline condition during the Holocene and late Pleistocene [1]. Therefore, to assess the importance of this finding, we explore how frequent or intense hurricanes might suppress acroporid growth for centuries, then reconstruct acroporid trajectories from other areas to determine if gaps are common elsewhere, and conclude by comparing this baseline with the historical decline in acroporid growth, providing insight into its cause and future trajectory.

### 4.1. Centennial-scale suppression of acroporid growth

Several features of acroporid habitat and life history suggest an adaptation to physical disturbance, with hurricanes being the main driver of population dynamics [10,24]. First, monospecific thickets of *A. palmata* are restricted to reef-crest and frontal zones at depths of less than 5 m, which coincide with the wave-breaking depth of low-intensity hurricanes (e.g. category 2 hurricanes generate 3 m waves that break in 5 m of water [15]). Second, such shallow acroporid populations are thus chronically exposed to fragmentation by waves, fostering a reliance on asexual reproduction [10]. Third, the rapid growth of cloned colonies and frequent low-intensity disturbance create ideal conditions for fragment propagation and habitat monopolization, potentially leading to a continuous cycle of disturbance and recovery [12]. An estimate of cycle length is given by the minimum age of acroporid genets whose long-lived clones can reach 800 years [50].

Although less intense hurricanes can maintain and augment growth trajectories, stochastic variations in hurricane frequency and intensity could produce anomalies that also suppress acroporid growth [52]. This is exemplified by the catastrophic impact of Hurricane Hattie, a category 5 hurricane with 320 km h^−1^ winds and a 4−6 m storm surge, which struck the Belize Barrier Reef in 1961 [53]. Surveys before and after the hurricane revealed severe damage over a 60 km belt centred on the hurricane track, with all shallow corals destroyed except for rare massive head colonies. The reef-front geomorphology was also flattened, with the destruction of spur-and-grooves and the deposition of skeletal traction carpets [53]. More than a decade later, the lack of recovery led Stoddart [54] to conclude that it would take a century to return to a pre-hurricane state, three to four times longer than his initial estimate.

As surmised by Stoddart, the complete destruction of acroporids during such an intense hurricane would create a dependence on sexual reproduction, significantly delaying recovery. The absence of standing colonies would curtail sexual fertilization success via the Allee effect and limit outcrossing with different genotypes to produce viable offspring [55]. Moreover, the accumulation of post-embryonic mutation in long-lived monoclonal populations may increase the frequency of fertilization failure in veteran genets, causing degeneration of life-traits associated with sexual reproduction and raising the risk of ecologically driven sexual extinction [56,57]. Together, these factors impair sexual recruitment and reproduction after intense hurricanes, predisposing acroporid reefs to protracted intervals of recovery.

Given this impairment, the potential for discontinuities in acroporid trajectories rests on the return period of reef-destroying category 5 hurricanes. Intense hurricane strikes at any particular location are statistically improbable, suggesting average return periods spanning hundreds of years. Estimates based on the short 150-year historical record are therefore unreliable [58]. In addition, return-period calculation is sensitive to the type of analysis, the choice of hurricane hazard variables and local reef fetch [58]. For instance, estimates using univariate metrics such as storm surge, wind speed, or precipitation yield return periods from 100 to 300 years [58]. However, basin-scale models which combine and synthesize these data over millennia predict return periods of 350−1000 years [59]. Such centennial-scale return periods for intense hurricanes thus confirm the potential for growth discontinuities within multi-millennial trajectories of reef development.

The recurrence of reef-destroying hurricanes coupled with impaired sexual reproduction is, therefore, consistent with a heterogeneous growth trajectory for acroporids. However, why would reef recovery be suppressed for hundreds of years? The reason may relate to the degree of substrate alteration caused by intense hurricanes. First, the deposition of skeletal traction carpets generated by colony fragmentation creates an unstable substrate for coral recruitment, until removed by subsequent storms or stabilized by encrusters [60,61]. Second, the destruction of corals and their geomorphic structures would create a flattened, barren substrate, eliminating the habitat of crustose corallines and other cohorts that facilitate recruitment [62,63]. Third, the import of sexual recruits from upstream sources would be limited by the short planktonic phase and dispersal distance of acroporids, increasing both the time required for recruitment and the chance of spat destruction by subsequent smaller storms [64,65]. Collectively, these processes represent a habitat recolonization requiring centuries—not a recovery taking decades—and are thus consistent with the centennial-scale gaps observed in the 5500-year trajectory of acroporid growth at Punta Maroma.

### 4.2. Regional acroporid trajectory

Despite the significant adversity posed by intense hurricanes, the growth trajectory at Punta Maroma shows that after several hundred years of absence, acroporids eventually recolonized their former habitat and resumed growth. The degree of growth constancy on any acroporid reef thus reflects local variation in hurricane frequency and intensity during its development, generating dissimilar acroporid trajectories among reefs. Any coincidence between trajectories would, therefore, require an alternative explanation.

**Figure 3.**
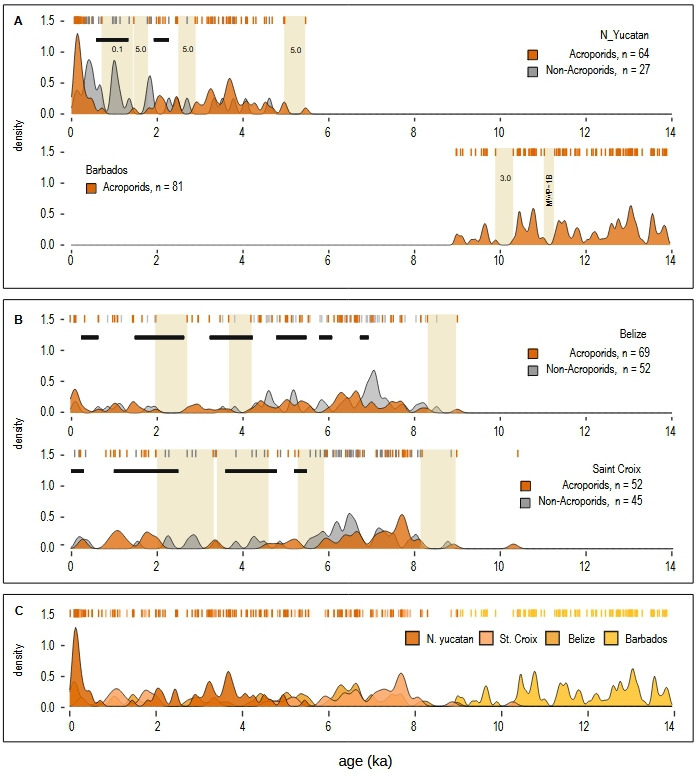
Reconstruction of acroporid growth trajectories across the Caribbean. (A) Centennial-scale gaps in high-resolution (<100 years) acroporid trajectories. The Punta Maroma trajectory has an 83-year resolution (64 U-series ages over 5300 years) and a statistically anomalous gap of 740 years (0.71−1.45 ka). Black horizontal lines denote intervals of high-hurricane frequency between 1.30−0.55 ka and 2.25−1.90 ka [51], which partly coincide with the youngest gap. The Barbados trajectory has a 59-year resolution (81 U-series ages over 4800 years) and a statistically anomalous gap of 360 years (9.92−10.28 ka). (B) Acroporid growth from low-resolution (>100 years) composite trajectories. The Belize trajectory has a 134-year resolution (67 U-series and calibrated 14C ages over 9000 years) from 20 sites over an approximately 200 km area [69]. The St Croix trajectory has a 210-year resolution (49 U-series and calibrated 14C ages over 10,200 years) from 14 sites over a 20 km area [67,68]. Gap integrity is uncertain given the low sample density at each site and high probability of undersampling; timing of growth is constrained by analytical age errors (<10%). Black horizontal lines denote high-hurricane-frequency intervals at St Croix [32,33] and Belize [70]. KDE bandwidth is the same as Punta Maroma data. (C) Regional growth composite from all high- and low-resolution sites showing acroporid growth constancy over the last 14 ka.

Several studies have reported gaps in acroporid growth using age data from multiple sites over large areas. For example, Shinn *et al.* [66] measured 31 radiometric ages of *A. cervicornis* clasts across the Florida Reef Tract and found two gaps (3.0 and 4.5 ka) in the last 6 ka. However, the subsequent inclusion of acroporid ages from local drill cores filled these gaps [30]. Similarly, Hubbard *et al.* [67,68] recovered 14 drill cores with 52 acroporid ages between 0.3 and 10.0 ka over a 20 km wide area off eastern St Croix (figure 3B). They combined these local data with published *A. palmata* ages from the Caribbean and found two gaps (5.9−5.2 ka and 3.0−2.2 ka), concluding they were consistent with regional episodes of disease or bleaching. Along the Belize Barrier Reef, Gischler *et al.* [69] also reported coincident gaps over the last 9 ka (6.0−5.5 ka, 4.2−3.7 ka and 2.7−2.0ka) from 20 cores with 127 ages (figure 3B). The problem with all of these large-area reconstructions is the low sample density at each site, which makes it impossible to exclude undersampling as a cause of gaps (electronic supplementary material, figure S3). Only the timing of acroporid growth has any certainty.

More localized acroporid trajectories with higher ecological resolutions have only been reconstructed at a few sites. For example, both Aronson *et al.* [71] and Greer *et al.* [72] reported up to 3 ka of growth constancy in lagoonal *A. cervicornis* thickets from Belize and the Dominican Republic. However, reefs protected from open-ocean conditions are subject to fewer disturbances, increasing the likelihood of growth constancy over time.

In addition to our densely sampled reconstruction from Punta Maroma, the only other open-ocean site where an acroporid trajectory can be reconstructed at an ecological resolution is Barbados (figure 3A). There, with the objective of reconstructing postglacial sea level, 81 acroporid ages were reported from a submerged 9−14 ka old reef-crest in Oistins Bay [73]. Further analysis of this core sequence by Blanchon *et al.* [74] showed frequent age reversals, indicating that the deposit contained hurricane-generated clasts. Our reconstruction of acroporid growth using these data in figure 3 shows a relatively continuous trajectory with a single 360 year gap between 10.0 and 10.35 ka. The probability of encountering a gap of this length with 81 samples is approximately 3%, suggesting that it probably represents an absence or low abundance of acroporid growth rather than undersampling (electronic supplementary material, figure S4C). However, given the core-based sampling protocol, it is still possible that this densely sampled reconstruction may not fully represent the acroporid growth trajectory at Barbados.

If the core-based sequence from Barbados is representative, then high-resolution acroporid trajectories from these sites allow us to draw two conclusions. First, both contain statistically anomalous gaps within an approximately 5 ka interval of reef-development which, in the case of Punta Maroma, coincides with a positive hurricane-frequency anomaly derived from an independent coastal-sediment archive [51]. Second, these centennial-scale gaps do not coincide with those at other sites (figure 3). For example, the two gaps at Punta Maroma between 700 to 1800 cal. years are filled by a growth interval at Buck Island on St Croix, regardless of its lower resolution. Similarly, gaps in lower-resolution reconstructions from St Croix are filled by growth at Punta Maroma and Belize (figure 3). The heterogeneity of acroporid growth among reefs is thus consistent with the stochastic spatio-temporal variation in hurricane frequency, which predicts that, although growth intervals might overlap, the gaps should not, given the improbability of simultaneous strikes by intense hurricanes.

### 4.3. Baseline and future trajectory

Our reconstruction of high-resolution acroporid trajectories from Punta Maroma and Barbados, and inclusion of lower-resolution trajectories from other sites (figure 3), provides a detailed Holocene baseline which places the historical decline of acroporids within a natural palaeoecological context. Several conclusions can be drawn from this Holocene record.

First, the presence of centennial-scale gaps in acroporid trajectories during the last 14 ka predicts that historical reefs have at least two ecological states: a ‘monopoly state’ where acroporids dominate the habitat, forming dense monospecific thickets on reef-crests exposed to open-ocean waves, and a ‘depleted state’ where acroporids are largely absent, and the habitat is dominated by other benthos. These two states represent reefs that have been either fully colonized by acroporids or require recolonization following destruction by an intense hurricane. Both are consistent with early descriptions of Caribbean reefs made before their rapid decline. For example, in the now classic description of Jamaican reefs, Goreau [5] contrasted the lush acroporid-dominated reef-crest and spur-and-groove zones along the north coast with the barren crests and absence of spur-and-groove along the south coast. He suggested these contrasting states represented a climax condition in the protected north and a regressive condition in the hurricane-prone south [5]. Reefs in a depleted state were also reported from the Alacran Reef complex, Mexico, by Kornicker & Boyd [75], from San Andres and Providencia Islands, Colombia, by Geister [6], and from the entire Eastern Caribbean by Adey & Burke [7]. Indeed, Adey & Burke [76] subsequently dated acroporids in drill cores from an eastern Martinique reef that gave a near-surface age of approximately 600 years, leading them to conclude that some may have been in a depleted state for hundreds of years.

Second, the centennial-scale gaps in high-resolution Holocene growth trajectories appear to be localized phenomena, with no regional-scale concordance (figure 3C). Although we cannot be fully certain of the integrity of all gaps or that core-based sequences fully represent acroporid growth trajectories, the timing of acroporid growth at each site is incontrovertible. Thus, the reconstruction composite shown in figure 3C confirms that acroporid growth has been regionally consistent across the Caribbean during the Holocene, which allows us to affirm that the regional decline in acroporids over the last 50 years is unprecedented over this interval.

Finally, the unprecedented scale of the historical decline in acroporids implicates anthropogenic factors as playing a significant and ongoing role. Nevertheless, our reconstructions suggest that the proximate cause of this decline is the naturally low rate of sexual recruitment in acroporids [77], which inherently impairs recolonization for hundreds of years following extreme disturbances. Human activities have merely added new types of extreme disturbance to natural ones, such as pollution-induced diseases [78] and warming-induced bleaching [29]. This has increased the frequency of local acroporid extirpation, shifting more reefs to a depleted state and increasing fragmentation and isolation of acroporid populations [38]. This coupling of impaired recruitment and population isolation implies recolonization of these sites by acroporids is unlikely for hundreds of years into the future, predicting a continuing decline as surviving reefs are eliminated one-by-one by future disturbance events, natural or otherwise [38,39]. Reversing this trend necessitates reducing the frequency of severe disturbance events, actively restoring acroporid populations on depleted reefs, and immediately protecting reefs where acroporids are still abundant.

## Data Availability

Data and relevant code for this research work are stored in GitHub [79] have been archived within the Zenodo repository [80]. Supplementary material is available online [81].
